# Relapsing Fevers: Neglected Tick-Borne Diseases

**DOI:** 10.3389/fcimb.2018.00098

**Published:** 2018-04-04

**Authors:** Emilie Talagrand-Reboul, Pierre H. Boyer, Sven Bergström, Laurence Vial, Nathalie Boulanger

**Affiliations:** ^1^Early Bacterial Virulence: Borrelia Group, Université de Strasbourg, Facultés de Médecine et de Pharmacie, CHRU Strasbourg, Fédération de Médecine Translationnelle de Strasbourg, VBB EA 7290, Strasbourg, France; ^2^Department of Molecular Biology, Umeå University, Umeå, Sweden; ^3^Laboratory for Molecular Infection Medicine Sweden, Umeå University, Umeå, Sweden; ^4^CIRAD BIOS, UMR15 CIRAD/Institut National de la Recherche Agronomique “Contrôle des Maladies Animales Exotiques et Emergentes,” Equipe “Vecteurs,” Campus International de Baillarguet, Montpellier, France; ^5^Centre National de Référence Borrelia, Centre Hospitalier Universitaire, Strasbourg, France

**Keywords:** relapsing fever, *Borrelia*, *Ornithodoros*, hard ticks, *Borrelia miyamotoi*, neurotropism, antigenic variations

## Abstract

Relapsing fever still remains a neglected disease and little is known on its reservoir, tick vector and physiopathology in the vertebrate host. The disease occurs in temperate as well as tropical countries. Relapsing fever borreliae are spirochaetes, members of the *Borreliaceae* family which also contain Lyme disease spirochaetes. They are mainly transmitted by *Ornithodoros* soft ticks, but some species are vectored by ixodid ticks. Traditionally a *Borrelia* species is associated with a specific vector in a particular geographical area. However, new species are regularly described, and taxonomical uncertainties deserve further investigations to better understand *Borrelia* vector/host adaptation. The medical importance of *Borrelia miyamotoi*, transmitted by *Ixodes* spp., has recently spawned new interest in this bacterial group. In this review, recent data on tick-host-pathogen interactions for tick-borne relapsing fevers is presented, with special focus on *B. miyamotoi*.

## Introduction

Tick-borne borreliosis are vector-borne diseases including Lyme disease, the most important tick-borne disease from the northern hemisphere, but also relapsing fevers (RF) especially prevalent in temperate and tropical areas (Ogden et al., 2014). Within tick-borne relapsing fevers (TBRF), the vectors are mainly argasid vectors also known as “soft ticks” of the genus *Ornithodoros*. Interestingly, some species are transmitted by ixodid vectors or “hard ticks.” The bacteria are all maintained in enzootic cycles with the human as accidental host except *B. duttonii* in Africa which seems strictly human and has no identified animal reservoir (Lopez et al., 2016). Whereas, Lyme disease continues to be extensively studied, TBRF, although known for ages, remain neglected diseases with only few studies elucidating the interactions between host, tick and pathogens.

In this review, we will present the most recent data on host/vector/pathogen interactions in soft tick-borne RF (STBRF) and in hard tick-borne RF (HTBRF), with a special focus on *Borrelia miyamotoi*, discovered recently as a human pathogen (Platonov et al., 2011). Concentrating on TBRF agents, the louse borne infection caused by *B. recurrentis* will not be further discussed. First, we will present the different pathogenic species of these spirochaetes and their characteristic adaptive strategies in the reservoir, the vector, and the host, as well as the phylogenetic evolution compared with their ecological features. Then, the vertebrate host/TBRF borreliae interactions of this zoonotic disease transmission system will be reviewed, with special interest in the human disease and mechanisms of immune evasion. We will describe the tick/TBRF borreliae interactions involved in the transmission of spirochaetes to vertebrates. Finally, we will provide an overview of the state of the art in HTBRF.

## Multiple pathogenic species of TBRF and adaptation to specific ecological niches

### Systematics and phylogeny of TBRF borreliae

TBRF are spirochaetes of the *Borrelia* genus within the family of *Borreliaceae* (Gupta et al., 2013). Recently, the *Borrelia* and *Borreliella* genera, which respectively contain the TBRF-associated species and the Lyme disease pathogens (lately denominated “TB-RF borreliae” and “LD borreliellae”) have been divided into two taxonomical groups. This distinction is based upon their nucleotide and protein signatures, their phylogeny of the 16S rRNA gene/conserved proteins and their phylogenomic metrics (Adeolu and Gupta, 2014; Oren and Garrity, 2015). Their arthropod vectors as well distinguish these two genera because schematically hard ticks transmit Lyme disease pathogens whereas soft ticks transmit TBRF pathogens. But this rule has some exceptions given that several RF agents are transmitted only by hard ticks (e.g., *B. miyamotoi*) and one species, *B. recurrentis*, is louse transmitted.

The taxonomy of TBRF borreliae species was historically based on a concept of co-speciation bacteria/tick (Wang and Schwartz, 2011), but the latest species descriptions rely on molecular methods (Fingerle et al., 2016). Currently, there are 22 validly published species names in the genus (Wang and Schwartz, 2011; Adeolu and Gupta, 2014; Table 1). Six other taxa were also proposed (“*B. merionesi*,” “*B. lonestari*,” “*B. microti*, “*Canditatus* B. texasensis,” “*Candidatus* B. algerica,” and “*Candidatus* B. kalaharica”) but none could be validated according to the current taxonomic rules, because the deposit of different strains in collections was not possible (Lin et al., 2005); indeed cultivation remains difficult for some species.

**Table 1:** Valid and proposed (in bold) species in the genus *Borrelia*.

***Borrelia* species (year of description)**	**Vectors**	**Geographical distribution**	**Hosts**	**Human diseases**	**References**
**OLD-WORLD RF BORRELIAE**					
***‘Candidatus* B. algerica’ (2015)**	Unknown	Algeria	Human	TBRF	Fotso Fotso et al., 2015
*B. baltazardii* (1979)	Unknown (*O. tholozani* in lab experiment only)	Iran	Human	TBRF, Thrombocytopenic purpura	Karimi et al., 1979; Naddaf et al., 2017
*B. caucasica* (1945)	*O. verrucosus*	Azerbadjan, Georgia, Armenia	Rodents, Human	TBRF	Maruashvili, 1945; Felsenfeld, 1965; Assous and Wilamowski, 2009
B. crocidurae (1917)	*O. sonrai*	Western and Northern Africa	Insectivores, Rodents, Human	TBRF, Mild symptomatology	Leger, 1917; Felsenfeld, 1965; Vial et al., 2006a; Trape et al., 2013
*B. duttonii* (1906)	*O. moubata* s.l. complex	East, Central and Southern Africa, Madagascar	Human	TBRF, Neurological signs, Ocular complications, Neonatal infections,	Novy and Knapp, 1906; Felsenfeld, 1965; Cadavid and Barbour, 1998; Larsson et al., 2006; Rebaudet and Parola, 2006
*B. graingeri* (1953)	*O. graingeri*	Kenya	Rodents, Human	Flu-like syndrome	Heisch, 1953; Felsenfeld, 1965; Wang and Schwartz, 2011
*B. harveyi* (1947)	Unknown	Kenya	Monkey	Unknown	Garnham, 1947, 1950
*B. hispanica* (1926)	*O. erraticus, O. marocanus*	Maghreb, Spain, Portugal, Greece, Cyprus	Rodents, Insectivores, Weasels, Foxes, Bats, Jackals, Dogs, Human	TBRF, Ocular complications, Neurological signs (rare)	Buen, 1926; Felsenfeld, 1965; Cadavid and Barbour, 1998; Trape et al., 2013
***‘Candidatus* B. kalaharica’ (2016)**	Unknow ‘likely a soft tick’	Southern Africa	Human	TBRF	Fingerle et al., 2016
*B. latyschewii* (1941)	*O. tartakovskyi*	Central Asia, Middle East	Rodents, Human	Flu-like syndrome	Sofiev, 1941; Baltazard, 1952; Goubau, 1984; Assous and Wilamowski, 2009
***‘B. merionesi*’ (1948)**	*O. merionesi, O. costalis*	Morocco	Rodent, Monkeys	No	Blanc and Maurice, 1948; Felsenfeld, 1965; Diatta et al., 2012; Trape et al., 2013
***‘B. microti*’ (1947)**	*O. erraticus*	Iran	Human	TBRF	Rafyi, 1947; Felsenfeld, 1965; Naddaf et al., 2012
*B. persica* (1913)	*O. tholozani*	Middle East, Egypt, Central Asia, India	Rodents, Dogs, Cats, Human	TBRF, Neurological signs (rare), Respiratory distress syndrome (rare)	Dschunkowsky, 1913; Cadavid and Barbour, 1998; Yossepowitch et al., 2012; Baneth et al., 2016
*B. recurrentis* (1874)	*Pediculus humanus*	Virtually worldwide, Currently Ethiopia, Sudan	Human	Louse-borne RF, Neurological signs	Lebert, 1874; Cadavid and Barbour, 1998; Hoch et al., 2015
*B. tillae* (1961)	*O. zumpti*	Southern Africa	Rodents	No	Zumpt and Organ, 1961; Felsenfeld, 1965
**New-World RF borreliae**					
*B. brasiliensis* (1952)	*O. brasiliensis*	Brazil	Human	TBRF	Davis, 1952; Lopez et al., 2016
*B. coriaceae* (1987)	*O. coriaceus*	USA	Rodents, Deer	Likely not pathogenic	Johnson et al., 1987; Nieto et al., 2012; Nieto and Teglas, 2014; Lopez et al., 2016
*B. dugesii* (1949)	*O. dugesi*	Mexico	Unknown	Unknown	Mazzotti, 1949; Wang and Schwartz, 2011
*B. hermsii* (1942)	*O. hermsi*	British Columbia (Canada), Western USA	Rodents, Deer, Dog, Human	TBRF, Neurological signs (rare), Neonatal infections (rare)	Davis, 1942; Cadavid and Barbour, 1998; Schwan et al., 2007; Centers for Disease Control Prevention, 2012; Nieto et al., 2012; Kelly et al., 2014; Nieto and Teglas, 2014
*B. mazzottii* (1956)	*O. talaje*	Mexico and Guatemala	Unknown	Likely TBRF	Davis, 1956; Lopez et al., 2016
*B. parkeri* (1942)	*O. parkeri*	Western USA	Rodents, Horses, Human	TBRF	Davis, 1939, 1942; Walker et al., 2002
*B. turicatae* (1933)	*O. turicata*	British Columbia (Canada), US, Mexico	Rodents, Dog, Human,	TBRF, Ocular complications, Neurological signs	Brumpt, 1933; Cadavid and Barbour, 1998; Schwan et al., 2005; Lopez et al., 2016
*B. venezuelensis* (1921)	*O. rudis*	Panama, Columbia, Venezuela, Ecuador, Paraguay	Unknown	TBRF	Brumpt, 1921; Goubau, 1984
**WORLDWIDE AVIAN RF BORRELIAE**					
*B. anserina* (1891)	*Argas* spp.	Worlwide	Birds	No	Sakharoff, 1891; Marchoux and Salimbeni, 1903; Fabbi et al., 1995; Hovind-Hougen, 1995
**HARD TICK-BORNE RF BORRELIAE**					
***‘B. lonestari* ’ (1996)**	*Amblyomma americanum* (lone star tick)	USA	Birds, Deers	No	Barbour et al., 1996; Jordan et al., 2009; Castellaw et al., 2011
*B. miyamotoi (1995)*	*Ixodes persulcatus, I. ricinus, I. scapularis*	Asia, Europe, USA	Rodents, Birds, Human	Flu-like syndrome, TBRF, Neurological signs	Fukunaga et al., 1995; Platonov et al., 2011; Gugliotta et al., 2013; Wagemakers et al., 2017
***‘Candidatus* B. texasensis’** (2005)	*Dermacentor variabilis* (American dog tick)	USA (Texas)	Unknown	Unknown	Lin et al., 2005
*B. theileri* (1903)	*Rhipicephalus* spp.	Africa, Australia, North, and South America	Cattle, Sheep, Goats	No	Laveran, 1903; Theiler, 1905; McCoy et al., 2014

**Figure 1:**
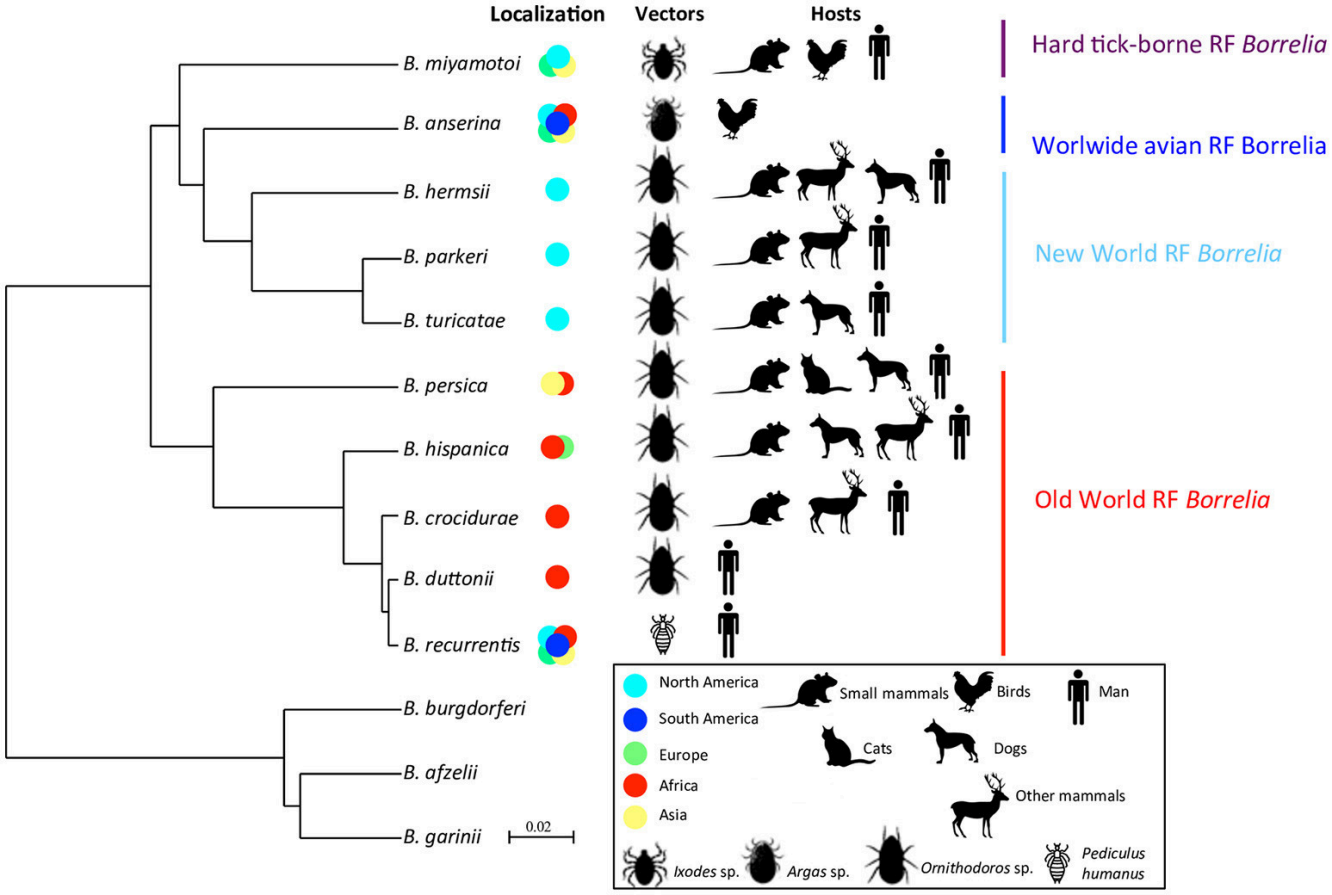
Phylodendrogram of Average nucleotide identity values among RF borreliae genomes. LD *Borreliella* genomes have been taken as outgroup. The phylodendrogram has been obtained by pairwise comparison of average nucleotide identity (ANI) between two genome sequences of each RF borreliae species sourced from Adeolu and Gupta (2014) and Elbir et al. (2014). Topology created on http://genomes.urv.cat/UPGMA/. The horizontal and vertical lines represent genetic distance, with the scale bar indicating 2% of difference from ANI = 100%. Other related species in SLPA or MLPA based on literature review are: (1) “*B. microti*,” “*B. merionesi*” and *Ca*. B. algerica in the Old world RF group (Naddaf et al., 2012; Trape et al., 2013; Fotso Fotso et al., 2015), (2) *B. coriaceae* and *Ca*. B. texasensis in the New world RF group (Fukunaga et al., 1996; Lin et al., 2005; Naddaf et al., 2012), and (3) “*B. lonestari*” and *B. theileri* in the Hard-tick-borne RF group (Lee et al., 2014; Hagen et al., 2018).

Although they harbor differences in their rates of evolution and robustness, several molecular chronometers of RF borreliae housekeeping genes (e.g., 16S rRNA, flagellin, glycerophosphodiester phosphodiesterase GlpQ) and non-coding sequences from the linear chromosome are quite congruent to delineate TBRF phylogenesis (Fukunaga et al., 1996; Ras et al., 1996; Scott et al., 2005; Oshaghi et al., 2011). Single gene phylogenetic analyses are supported by studies of multiple loci (2 to 7 among *rrs, flaB, glpQ, groEL, p66, recG*, and 16S−23S rRNA intergenic spacer IGS) (Toledo et al., 2010; Trape et al., 2013; Naddaf et al., 2017), extended multilocus phylogenetic analysis (MLPA) panel of 25 conserved coding DNA sequences (Adeolu and Gupta, 2014) and phylogenetic analysis based on 266 sets of single-copy orthologues present in all genomes (Di et al., 2014). According to the highest resolutive methods, TBRF borreliae embrace four lineages also harboring common ecological features, including a vector of *Ixodidae* (“Hard-ticks”) or *Argasidae* (“Soft-ticks”) family and/or geographic distribution: (1) Old-World TBRF borreliae, (2) New-World TBRF borreliae, (3) the worldwide avian TBRF borreliae (i.e., *B. anserina*) and (4) the HTBRF group (Table 1; Adeolu and Gupta, 2014; Di et al., 2014). Other borreliae species may be virtually attached to these phylogenetic groups by analysis of SLPA/MLPA-based studies mostly preserving the ecological specificities of each clade (Figure 1). Phylogenetic positions of some recent strains retain ambiguities after the sequencing of too few genes due to incongruities between genes (“*Ca*. B. kalaharica” and a new clinical *Borrelia* sp. in Iran) (Fingerle et al., 2016; Naddaf et al., 2017) and would require additional phylogenetic analysis to clarify relationships between lineages. In addition, several TBRF borreliae species could not be included in any phylogeny comparison because no DNA sequences have been available so far (*B. venezuelensis, B. caucasica, B. harveyi, B. dugesii, B. braziliensis, B. graingeri, B. mazzottii, B. tillae*, and *B. baltazardii*).

Concerning the species level, African RF borreliae (*B. crocidurae, B. duttonii*, and *B. recurrentis*) are closely related species and may correspond to the ecotypes of a unique genomospecies regarding their average nucleotide identity (ANI > 96%) as suggested by Elbir et al. (2014), corroborating the genomic deep-analysis concluding that the genome of *B. recurrentis* was a degraded subset of *B. duttonii* (Lescot et al., 2008). Similarly, the North American *B. parkeri* and *B. hermsii* are also very closely related species regarding their ANI values (Adeolu and Gupta, 2014). Up to now, the classification of RF borreliae as presented herein remains official (Table 1) but may probably be further revised due to the taxonomically admitted bacterial concept of “genomic species” or genospecies as suggested by several authors (Ras et al., 1996; Wang and Schwartz, 2011; Elbir et al., 2014). The harmonization of RF borreliae taxonomy within current rules and the recognition of ecotypes could be achieved by re-classifications of the species and subspecies levels. Nevertheless, the current systematics of RF borreliae reflects very well the characteristics of vector-bacteria-host association of this genus that can be considered as a remarkable case-study of adaptation in correlation with the bacterial concept of “ecotype species” whatever the name of the taxonomic level. The availability of new completed genome sequences as well as genetic population studies of strains from different origins may in the coming years, increase the understanding of the phylogeny of RF borreliae and also clarify taxonomic issues.

### Bacterial features and vector/host associated lifestyles

RF borreliae are motile, chemo-organotrophic, microaerophilic and host-associated bacteria (Kelly, 1971; Barbour and Hayes, 1986; Adeolu and Gupta, 2014). These spirochaetes dwell extracellularly in ticks as well as in the blood and organs of their vertebrate hosts. They usually grow at temperatures between 33 and 35°C corresponding to mammalian host temperature, but they are also able to multiply at 22°C (tick temperature) as proven *in vitro* for *B. turicatae* (Wilder et al., 2016). Genes encoding enzymes for the synthesis of most amino acids, fatty acids, enzyme cofactors, and nucleotides are absent in the RF borreliae genomes as shown in LD borreliellae (Fraser et al., 1997; Adeolu and Gupta, 2014). The *Argasidae* and *Ixodidae* cuticle, which contains chitin derived from the polymerization of N-Acetyl Glucosamine (NAG), might be an important nutrient source for *Borreliaceae* during the arthropod-associated phase (Hackman and Goldberg, 1985; Tilly et al., 2001).

The genome size of RF borreliae (1–1.5 Mb) is smaller than the one in other pathogenic bacteria which have a more versatile lifestyle (e.g., *P. aeruginosa*, 6.3 Mb). Evolution by genome reduction is well correlated to the degenerated lifestyle of several well-adapted pathogens (Dobrindt and Hacker, 2001), that are likely to include RF borreliae which have a restricted niche in the vector and in the vertebrate host. Correlating to their size, RF borrelia genomes have a limited repertoire which very well reflects their adapted lifestyle, including only few virulence-associated genes (Fraser et al., 1997; Adeolu and Gupta, 2014). These genomes harbor a linear megaplasmid of 160 kb having a fairly conserved synteny among *B. duttonii* (lp165), *B. hermsii* (lp174), and *B. turicatae* (lp150) strains which is not found in LD borreliellae (Miller et al., 2013). The *B. turicatae* megaplasmid is subject to a shift in its transcriptional profile between *in-vitro* tick-like growth conditions and murine infected blood, identifying a cluster encoding for putative surface lipoproteins likely involved in vector colonization and host-vector interactions (Wilder et al., 2016).

### Geographic distribution

The four main species responsible for STBRF in Europe are: *B. hispanica, B. persica, B. caucasica* and *B. crocidurae* (Rebaudet and Parola, 2006). In Africa, the main circulating bacteria are *B. crocidurae* in Western and Northern Africa, *B. duttoni* in Eastern, Central and Southern Africa and *B. hispanica* in the Maghreb (Felsenfeld, 1965; Rebaudet and Parola, 2006; Vial et al., 2006a; Trape et al., 2013). In the United States, three predominant species have been identified and were particularly studied in the western part: *B. hermsii, B. parkeri*, and *B. turicatae* (Barbour, 2005; Sonenshine and Roe, 2013). The STBRF borreliae species of African (*B. hispanica, B. crocidurae*, and *B. duttonii*) and American clades (*B. hermsii, B. parkeri*, and *B. turicatae*) have evolved in narrow geographic areas, probably due to the presence of specific reservoirs and arthropod vectors. In Iran, several species are associated with human STBRF cases, including “*B. microti*,” *B. persica* and another genotype close to the complex *B. duttonii*/*B. recurrentis* by 16S−23S rRNA intergenic spacer sequence (Naddaf et al., 2015).

In HTBRF, the distribution of the particular species is not limited to a single continent, since besides Asian *Borrelia* sp. (“*lonestari*-like”) and American borreliae (“*B. lonestari*,” “*Candidatus* B. texasensis”), worldwide spread taxa (*B. miyamotoi, B. theileri*) are also found (Table 1).

## Vertebrate host-RF borreliae interaction

### Host specificity

Vertebrate host specificity is variable between RF *Borrelia* spp., since most of the species can infect small mammals and human, and some species can infect birds, domestic or wild mammals without a clear lineage specificity (Table 1, Figure 1). Inversely, strong association has been described between RF borreliae and the vector species, to such an extent that some authors proposed soft tick vectors as the original natural reservoir for RF borreliae (Barbour, 2005). Regarding the nidicolous or endophilous lifestyle of soft ticks, which colonize sheltered underground habitats like nests, burrows, caves or cracks inside buildings (Sonenshine, 1993; Vial, 2009), it seems obvious that their vertebrate hosts are likely to be rodents or other small vertebrates directly present inside or around the habitats. Such preference for peculiar habitats may drive the host specificity of ticks and their associated RF borreliae. RF borreliae-tick-vertebrate host interrelationships were summarized as follows by Hoogstraal (1979): “*Borrelia* develops as symbiont of soft ticks but act as parasites in mammals and birds, which serve as borrelial reservoirs and amplifiers following bites by infected ticks.”

However, two remarkable host associations are reported for RF borreliae. The spirochaete *B. anserina* has a host restriction in birds. It was experimentally proven to be infectious to chicken and various birds but not to rodents and non-human primates (McNeil et al., 1949; Lisbôa et al., 2009). In parallel, the RF borreliae of the *B. duttonii*/*B. recurrentis* complex has only been associated with human cases up to now, although *B. duttonii* can infect chicken and rodents under laboratory conditions (Kervan, 1947; Yokota et al., 1997; Larsson et al., 2009). The DNA of *B. duttonii* has also been detected in the blood of domestic chickens and pigs in Tanzania (McCall et al., 2007).

### Host sensitivity

The characterization of RF borreliae has been conducted for a long time through experimental infection of laboratory animals such as the mouse, the rat, the rabbit, the guinea pig or the monkey, and through the description of resulting pathogenicity (Goubau, 1984). However, not all the infections do automatically result in the death of the animal, and symptoms can vary depending on the inoculated bacterial load as well as the age and the metabolism of the animal (Hindle, 1935). Likewise, some mammals have been found naturally infected by RF borreliae, without any clinical signs and during a long time, and are supposed to be vertebrate reservoirs of RF borreliae (Rhodain, 1976). Several rodent species, squirrels, foxes, shrews, porcupines, opossums, armadillos, and also some domestic animals, have been mentioned as possible reservoirs worldwide (Hindle, 1935; Barbour, 2005). Their bacteraemia is very low and cannot be detected by classical thick blood smear, to such a point that Burgdorfer and Baltazard (1954) classified these infections as “occult.” Only the inoculation of blood or organ homogenate, such as brain or spleen, to sensitive animals can lead to sufficient and detectable amplification of RF borreliae. Due to the proximity of rodents to human habitations, especially in rural areas, such organisms may be the most important carriers for RF borreliae (Hindle, 1935). In the United States, pine squirrels and chipmunks are considered major reservoirs for RF borreliae (Trevejo et al., 1998; Paul et al., 2002; Fritz et al., 2004). However, in West Africa, Mathis et al. (1934) noticed that the insectivorous formerly called *Crocidura stampfii* showed high spirochetemia in blood and suggested that it could be a better reservoir than small rodents for *B. crocidurae*, since soft ticks were more likely to become infected through biting such animals.

### Reservoir host dynamics and RF borreliae transmission

The specificities of small mammal reservoirs, as well as those of soft tick vectors, supposedly the original hosts for RF borreliae, can influence the transmission dynamics of such pathogens to humans, who remain an occasional host. Host-vector-pathogen interactions here will focus only on RF borreliae transmitted by *Ornithodoros* soft ticks since they are the most investigated worldwide.

After their contamination through biting on infectious vertebrate reservoirs, *Ornithodoros* ticks can remain infected for several weeks, months, or years (Hindle, 1935; Gaber et al., 1984; Goubau, 1984). Because of multiple blood feeding along their life cycle and a very long lifespan of adult development stages (Morel, 2003; Gray et al., 2014), soft ticks have many occasions to transmit borreliae. Their blood feeding is short, from a few minutes to a few hours for most stages, except for larval stages of the former genus *Alectorobius* (e.g., *O. talaje*) that feed for several days (Loomis, 1961; Morel, 2003), which results in very short parasitic phases on their vertebrate host. Furthermore, these ticks are typically nidicolous or endophilous and photophobic, thus predestined to live in the underground and sheltered microhabitats (Sonenshine, 1993; Vial, 2009). Both features may lead to very low tick dispersal, as suggested by previous genetic or biological studies (Chabaud, 1954; Vial et al., 2006b). Small mammals being reservoirs of TBRF and hosts for ticks are also considered very sedentary because of their territorial behaviors (Powell, 2000). In view of these specificities, RF borreliae transmission is expected to occur locally. This was actually reported in the United States (Trevejo et al., 1998; Dworkin et al., 2002; Paul et al., 2002; Schwan et al., 2003; Fritz et al., 2004), Israel (Sidi et al., 2005), and in Jordan (Babudieri, 1957), where patients are mainly exposed to RF borreliosis during their visit and sleeping in rustic cabins or caves, which are colonized by infected ticks and rodent reservoirs. In Africa, local transmission of RF borreliae is also observed, and patients are generally contaminated in their own houses especially in rural areas where rodents commonly burrow undisturbed inside buildings (Cutler, 2006; Vial et al., 2006a).

**Figure 2:**
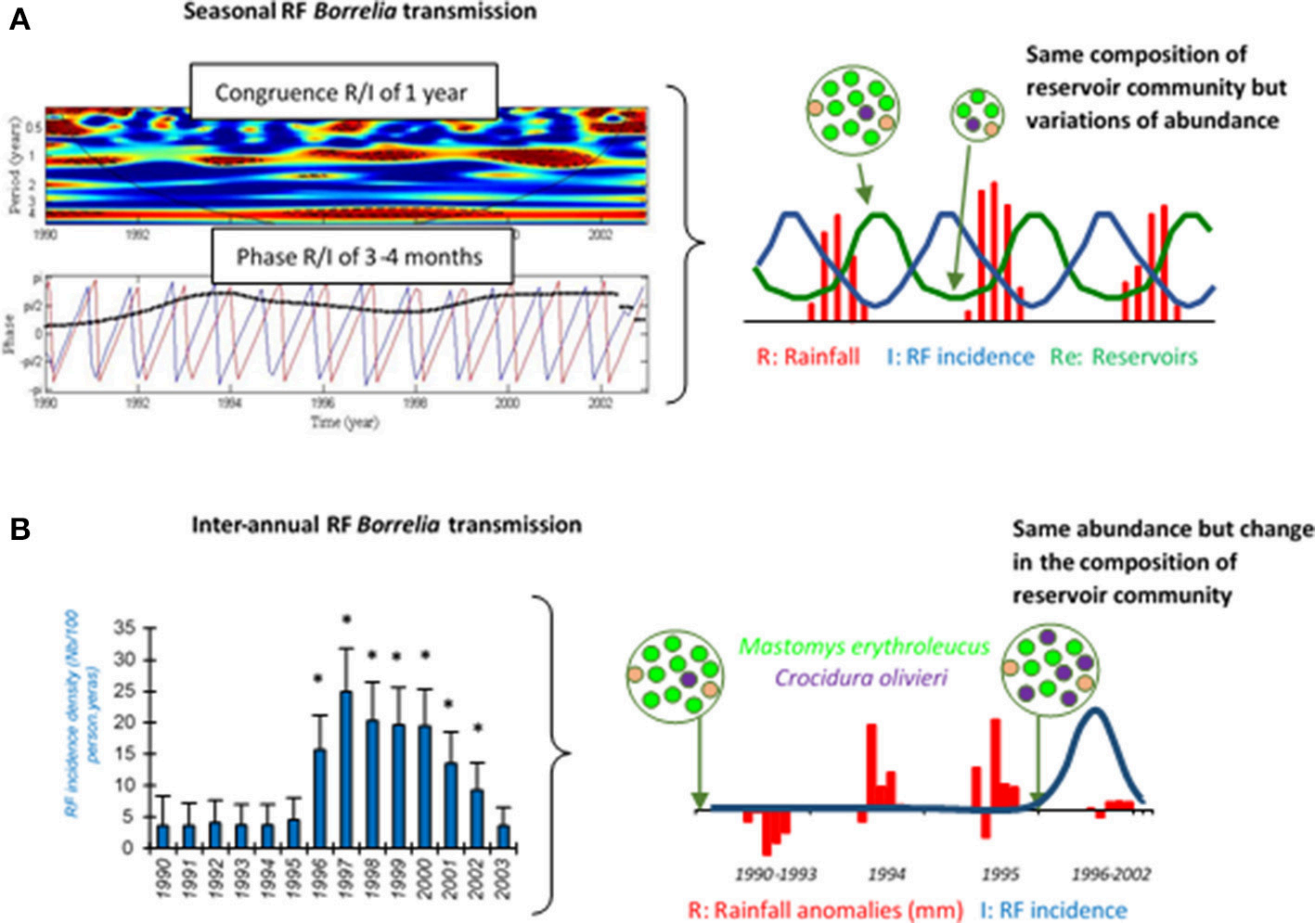
Seasonal **(A)** and inter-annual **(B)** RF borreliae transmission to human in Dielmo village, Senegal, from 1990 to 2003. In **(A)** left panel, the medium red shape in the first graph indicates the congruence between RF borreliae transmission and seasonal rainfall, and the black dotted line in the second graph indicates the 3–4 month phase between both time-series (RF borreliae transmission in blue and rainfall in red). In **(B)** left panel, the annual incidence density of RF borreliosis is expressed as the number of new infections per 100 persons per year, 95% confidence intervals are indicated and significant differences of incidence are reported by stars (*). In right panels, the schemes expose the likely interpretations for seasonal and inter-annual RF incidence variations, respectively, in relation to the dynamics of the small mammal reservoirs. The reservoir community is represented by a circle, including several dots of different colors as the different small mammal species, and the relative number of dots reflects the relative abundance of each species.

Interestingly, a longitudinal monitoring study of human borreliosis over 14 years in Dielmo village, Senegal, highlighted a spatial clustering of cases in and close to compounds where *O. sonrai* ticks were systematically collected, 10 years apart (Vial et al., 2006a). During this period, ticks seem to have lightly dispersed to a third site, which resulted in a new temporary focus of TBRF cases (Vial et al., 2006a). The compound-specific effect was significant on RF borreliosis incidence (*p* < 0.01 using random-effect Poisson regression). TBRF transmission to humans should show a seasonal pattern, corresponding to seasonal dynamics of rodent and tick populations. In Dielmo, Senegal, infections with TBRF rose throughout the year, but were actually most common in March and least common in October (Vial et al., 2006a). A wavelet analysis, which can be used to perform a timescale decomposition of ecological or epidemiological time series (Cazelles et al., 2014), showed a strong congruence between rainfall annual cycles and TBRF human cases, with a phase of 3–4 months (Figure 2A, left panel). This indicates that maximum transmission of TBRF to humans occurred 3–4 months before the rainy season, meaning at the end of the dry season when rodent reservoir populations decrease because of annual mortality related to the scarcity of food resources (Sicard and Fuminier, 1996; Figure 2A, right panel). *Ornithodoros* tick ecology could explain this non-intuitive result. Because of their endophilous lifestyle, tick populations are only slightly influenced by external climatic variations especially in subtropical regions, and it is accepted that the probability of tick biting inside one habitat should be more or less even along the year. In addition, *Ornithodoros* ticks do not show any host preferences, as a possible adaptation to host scarcity in their habitats (Morel, 2003; McCall et al., 2007; Palma et al., 2013). Consequently, ticks may mainly engorge on rodents when they are available in abundance directly in tick microhabitats, leading to a lower transmission of RF borreliae to humans due to lower tick biting pressure; inversely, when rodent populations decline, ticks may preferentially engorge on humans and RF borreliae transmission conjointly increases (Figure 2A, right panel). Apart from endemic TBRF in the rural African environment, seasonal RF borreliae transmission can also occur but it may then be rather due to human habits, with higher transmission during summer when people settle in tick-infested locations for holidays or increase their outdoor activities (Fihn and Larson, 1980; Dworkin et al., 2002; Masoumi Asl et al., 2009; Moemenbellah-Fard et al., 2009).

In temperate regions, *Ornithodoros* ticks can suffer from low temperatures during winter although they remain protected by their sheltered habitat, and can consequently stop questing and feeding (Skruinnik, 1939; Kadatskaia and Shashnikova, 1963; Oleaga-Pérez et al., 1990; Morel, 2003), which may result in a decrease of RF borreliae transmission.

Some authors indeed reported human RF outbreaks, which might not be a simple consequence of local transmission or improved surveillance, but highlighting a real increase in RF borreliae transmission. Factors sparking such outbreaks are always a matter of speculation because it is not possible to monitor them before first evidence of the impact. At the North Rim of the Grand Canyon, United States, populations of rodent hosts for *O. hermsi* were drastically reduced in 1973, possibly due to epizootic plague, and might have resulted in higher tick biting on humans and higher RF borreliae transmission; the same pattern was also observed in 1990 although there was no documented epizooty of plague (Paul et al., 2002). As already demonstrated for Lyme disease, biodiversity loss in small mammal reservoirs can contribute to promoting RF borreliae transmission based on the general concept of a dilution effect (Ostfeld and Keesing, 2000; LoGiudice and Gosain, 2003). In Dielmo village, Senegal, a RF outbreak was reported from 1996 to 2002, with two to six times more cases than previously (Vial et al., 2006a; Figure 2B, left panel). Small mammal captures conducted inside houses before and after these outbreaks in 1990 and 2002, revealed changes in the composition of the rodent community with the partial replacement of the common rodent species *Mastomys erythroleucus* by commensal shrews *Crocidura olivieri* (Figure 2B, right panel). RF borreliae detection in captured animals revealed higher prevalence of infection in *C. olivieri* than in any other rodent species, suggesting that this shrew species is a very competent reservoir for RF borreliae (Vial et al., 2006a). Other authors (Mathis et al., 1934; Burgdorfer and Mavros, 1970; Nieto and Teglas, 2014) also proposed this assumption of differential reservoir ability for maintaining RF borreliae between the different small mammals. Since *Ornithodoros* ticks have no host preference, they may have engorged proportionally much more on *C. olivieri* than on others and this might have resulted in higher tick infection and increased RF borreliae transmission to humans (Figure 2B, right panel). The relatively high longevity of *C. olivieri* (2–3 years) and its competitiveness against other commensal rodents (Churchfield, 1990) are both features that could favor persistent outbreaks for several years. Inversely, *M. erythroleucus* like many rodents is an annual species that depends on climatic conditions, with possible pullulating events due to abundant rainfall and vegetation growth (Sicard and Papillon, 1996). This might be the case for 1993–1994 (Figure 2B, right panel). Such abnormal population increase is usually followed by density-dependent crash (Leirs et al., 1997), leading to empty suitable niches for the development of *C. olivieri*.

### Human-*Borrelia* interactions

#### Clinical manifestations

One of the major difficulties in RF diagnosis and investigation of medical history is the variable disease presentation, i.e., variable clinical manifestations. The outcome may also be very different with a more or less asymptomatic presentation or a lethal progression, but it is usually severe among small children (Southern and Sanford, 1969). The feeding behavior of Argasid (soft) ticks, i.e., short period of feeding and the fact that the patients do not recognize the tick bite makes it hard to perform a correct clinical diagnosis. Although variable, the incubation time is about 1 week, i.e., between tick bite and the first manifest symptoms (Southern and Sanford, 1969), which result in high fever in the temperature range between 38.7 and 40°C (or even 41°C). The first fever period is usually the longest and lasts for about 4–7 days (Bryceson et al., 1970; Felsenfeld, 1971). This initial febrile episode is followed by a series of relapses (9 to 13 in STBRF), corresponding to peaks of spirochaetemia interspaced by a few days of remission. This is usually described during the course of non-fatal infections in absence of antibiotic treatment (Cutler, 2015).

The typical symptoms are flu-like with malaise and general ache, often involving myalgia and headache. Very often patients experience nausea with vomiting or diarrhea. Patients may also display different types of skin rashes that might have petechial or haemorrhagic manifestations. In mouse model, many of the Old-World RF species can bind to erythrocytes and generate cell aggregates that disrupt the microcirculation. These aggregates then create micro-thrombosis in arterioles anywhere in the body that affects the blood flow during spirochetemia (Shamaei-Tousi et al., 1999). During the spirochetemic peak, more or less severe bleeding can be observed. In cases with more severe haemorrhagic phenomena, bleeding from different organs may be seen, including nose bleed, haemoptysis, bloody diarrhea, haematuria. During severe RF borreliosis, hemorrhages may also be seen of the retina and cerebrum as well as bleeding into the sub-arachnoid space. Internal organs, such as the liver may also be affected, which can cause enlargement and tenderness that can be followed by jaundice. Relapsing fever may also affect the spleen causing micro-abscesses, enlargement and rupture. This systemic disease often causes respiratory symptoms with a cough as well as myocarditis further proving the difficulties to establish a differential diagnosis from influenza virus infection. The disease can resolve itself in the absence of treatment but mortality (around 5%) is observed in epidemics (Rebaudet and Parola, 2006; Ogden et al., 2014).

#### Severe presentations

Neurological symptoms are common during RF, but the presentation and severity of symptoms are variable, with infections caused by *B. duttonii* and *B. turicatae* being the most neurotropic. The most common symptoms are meningitis and facial palsy. Many RF cases with neurological involvement, including fatal ones, encompass sequelae with oedema and subarachnoid and parenchymal brain hemorrhages (Judge et al., 1974; Salih et al., 1977; Ahmed et al., 1980). Ocular complications have been reported as well (Cadavid and Barbour, 1998). In addition, depending on the infecting strain and changing with the phases of the disease, several hematological effects can be seen. Among those, a striking and pronounced thrombocytopenia as well as low hemoglobin and erythrocyte counts can be seen. If RF borreliosis remains untreated, a progressive waning of the general condition can occur at later stages, which is often accompanied with severe weakness and weight loss.

#### Penetration of tissues and barriers

*Borrelia* spirochaetes are known to readily penetrate biological barriers, which can be partly attributed to their helical shape and movement. Thus, flagellar mutants affect their helical shape and movement resulting in a deficient penetration through endothelial layers (Sadziene et al., 1991). However, the use of the host protease system to penetrate biological barriers during infection has been demonstrated in several studies. No endogenous protease has so far been attributed to *Borrelia* spirochaetes (Klempner et al., 1996), but they have the capacity to bind factors of the plasminogen activation system (PAS) such as the plasminogen (plg) and plg-activator. Klempner and coworkers revealed that the binding of human serine proteases can initiate a proteolytic activity that will be helpful for the efficient invasion during RF borreliosis (Klempner et al., 1996). Several *in vitro* and *in vivo* studies show the importance of the PAS during infections caused by *Borrelia* spirochaetes. These studies revealed that it was involved in the degradation of several substrates (Coleman and Benach, 2000) and also enhanced spirochaetes penetration through the endothelial cell layers (Coleman et al., 1995). In addition, the importance of the PAS was further shown in a series of animal experiments using plasminogen knockout mice (*plg*^−/−^). These *in vivo* studies revealed that there was a delay of RF borreliae spreading to tissues when the PAS is absent. This effect on penetration and invasion also reduced the bacterial amount in both brain and heart of infected animals (Gebbia et al., 1999; Nordstrand et al., 2001). However, the PAS activity is not the only mechanism in place as there are also indications that there is an additional activation of matrix metalloproteinases (Gebbia et al., 2001). Thus, several host proteases enable the dissemination through biological barriers, but the activity is not a critical factor for initially reaching the circulation, since the *plg* ^−/−^ knockout mice develop, although delayed, spirochaetemia similar to wild-type mice (Gebbia et al., 1999).

#### Relapsing fever in pregnancy

Reports from international organizations claim that approximately 10–15% of neonatal deaths in the World are caused by serious infections (Oza et al., 2015; United Nations Inter-agency Group for Child Mortality, 2017). A part of those infections is possibly caused by RF *Borrelia* in endemic regions. The consequences of RF borreliosis on pregnancy and subsequent pregnancy complications can either be mild with a slight decrease in birth weight and preterm delivery or severe resulting in miscarriage or neonatal death (Goubau and Munyangeyo, 1983; Brasseur, 1985; Melkert and Stel, 1991; Dupont et al., 1997; Jongen et al., 1997; van Holten et al., 1997). Interestingly, Dupont and coworkers reported that in Congo, more than 6% of pregnant women seeking healthcare were diagnosed with RF borreliae (Dupont et al., 1997). Several studies indicated that RF infection might be more severe during pregnancy, which is true for many infections (Goubau and Munyangeyo, 1983; Melkert, 1988). Still, no clear evidence has been presented supporting this statement. In contrast, Fuchs and Oyama published a study in which the mother had an uncomplicated mild RF borreliosis infection with low-grade fever, 3 weeks before labor but with a child fatal outcome only 36 h after delivery (Fuchs and Oyama, 1969). In a pregnancy animal model, it was revealed that RF borreliae can infect the fetus *in utero*. In this model, the *B. duttonii* infection can result in intrauterine growth retardation as well as placental damage with inflammation. Spirochaetes efficiently cross the maternal-fetal barrier, causing an infection of the fetus. It was shown that over 70% of fetuses became infected in the uterus of *B. duttonii* infected mice (Larsson et al., 2006). These infected mice exhibited noticeable intrauterine growth retardation, possibly caused by the histologically observed placental damage and inflammation. In addition, the impaired fetal circulation caused by spirochaete and erythrocyte interactions (see below) as well as lowered maternal hemoglobin was causing the described pregnancy complications in addition to the actual bacterial invasion of the placenta (Larsson et al., 2006).

#### Antigenic variations

In response to an exogenous change such as vertebrate immune response, a microorganism can modify its immunodominant antigens by a switch in gene expression of multiphasic surface proteins. RF borreliae represent a well-studied model for these antigenic variations. Once spirochetemia is high, RF borreliae are neutralized by IgM antibody immune response. A small part of bacteria harboring less prevalent antigens can escape the immune system and rises to cause a spirochetaemic relapse (Barbour and Restrepo, 2000; Alugupalli et al., 2003a).

**Figure 3:**
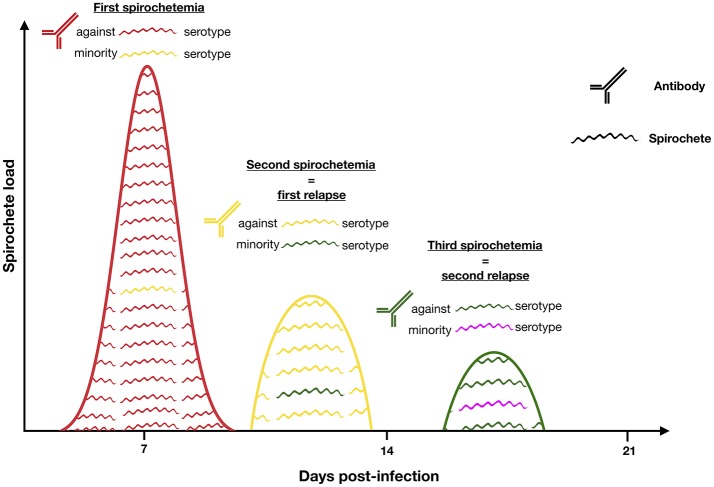
RF increases its persistence in blood by shifting the surface protein Vmp. When antibodies are mounted against the initial serotype (red) all bacteria expressing it are killed by Vmp-specific antibodies. Only those that have shifted to another serotype (yellow) survive and multiply to cause the first relapse. This battle continues until the host dies or the bacteria are eradicated from the blood. Antigenic variation is the mechanism causing the recurring fever which gave the disease its name. Remember that relapses rarely consist of one, single serotype.

Asymptomatic incubation in human RF borreliosis is estimated at 3–10 days, probably depending on the 4–5 h generation time of spirochaetes in the blood (mouse model) (Crowder et al., 2016). Then, an initial febrile episode followed by a series of relapses (3–5 in LBRF and 9–13 in TBRF) interspaced by a few days remissions are usually described during the course of non-fatal infections in absence of antibiotic treatment (Cutler, 2015). Schematically, each relapse corresponds to the rise of a new immunogenic variant of RF borreliae harboring a changed “Variable major protein” (Vmp) on its surface (Figure 3). There are two different families of Vmps, the Variable large proteins (Vlps) of ≈40 kDa and the Variable small proteins (Vsps) of ≈20 kDa. Both Vlps and Vsps families are encoded on linear plasmids (Hinnebusch et al., 1998). Each genome contains a collection of silent 600 bp *vsp* and 100 pb *vlp* copies on linear plasmids, while only one duplicate copy of these archived genes is transcriptionnaly active in a unique telomeric expression site on the same or another linear plasmid. Several mechanisms of gene conversion, DNA rearrangements, hypermutations and change in transcription locus appear to be involved in the replacement of the active *vsp* or *vlp* gene (Meier et al., 1985; Plasterk et al., 1985; Penningon et al., 1999; Dai et al., 2006; Raffel et al., 2014). The repertoire of Vmp encoding genes is highly diverse with 27 (17 *vlp*/10 *vsp*), 59 (38/21) and 82 (68/14) silent cassettes detected in the genomes of *B. recurrentis, B. hermsii*, and *B. duttonii*, respectively (Dai et al., 2006; Lescot et al., 2008).

The mechanism of antigenic variation is likely to be a common feature in all the different RF borreliae, since several orthologs of silent cassettes have been found. Up to now, these multiphasic changes have been demonstrated in the following species: *B. hermsii* (Plasterk et al., 1985), *B. turicatae* (Ras et al., 2000), and more recently in *B. miyamotoi* (Wagemakers et al., 2016).

Vsps and Vlps are phylogenetically related to the LD borreliellae surface proteins OspC (Outer Surface protein C) and VlsE (vmp-like sequence E), respectively (Zhang et al., 1997; Zuckert et al., 2001). In mammals, OspC is involved in the early phase of infection and in replacement VlsE is expressed later for the host immune evasion (Steere et al., 2016).

Beyond the host immune evasion, *in vivo* antigenic variations of *B. turicatae* are correlated to the emergence of different populations of serotypes well adapted to particular organs (e.g., NCS or joints) only depending on the expression of their surface Vmp (Cadavid et al., 1994). These distinctions in tissue tropism for 2 isogenic but antigenically distinct serotypes could be explained at least in part by differences in the ability of Vsps to bind extracellular matrix molecules of the host in link with their respective electrostatic surface properties (Magoun et al., 2000; Lawson et al., 2006). Similar results arguing for the existence of Vmp-related pathotypes are also reported among *B. hermsii* isogenic serotypes and by Vmp mutant experiments (Mehra et al., 2009; Raffel et al., 2014). In addition, Vmps of *B. recurrentis* expressed in the host can act as major TNF-inducing factors likely involved in extremely serious Jarisch-Herxheimer reaction following antibiotic treatment of louse-borne relapsing fever (Vidal et al., 1998).

#### Persistence

Borreliae spirochaetes are well adapted to persist as long as possible in a susceptible host, thus increasing the possibility of transmission to a naïve host by an arthropod vector. RF borreliae are blood-borne pathogens and when entering a blood vessel they will momentarily be transported to any blood perfused tissue. At the distal ends of the circulatory system, the blood flow is reduced and the speed of transportation of the spirochaetes will be decreased, resulting in a transmigration and invasion of spirochaetes through the endothelium into neighboring tissues and organs. *In vivo* studies on neurotropic RF *Borrelia* species revealed that these species could persist for a long time in the brain without causing any harm in the infected host. Neither any relapsing fever symptoms nor any spirochaetes in the blood were detected indicating a silent infection (Cadavid and Barbour, 1998; Larsson et al., 2006). This phenomenon of silent and persistent infections has not been proven in humans yet. But Cutler (2006) reported a low *B. duttonii* spirochaetemia in the blood of apparently healthy people in a village in Tanzania, demonstrating the importance and ability of RF borreliae to hide in immune privileged sites and cause silent infections.

#### Interactions in the circulation

**Figure 4:**
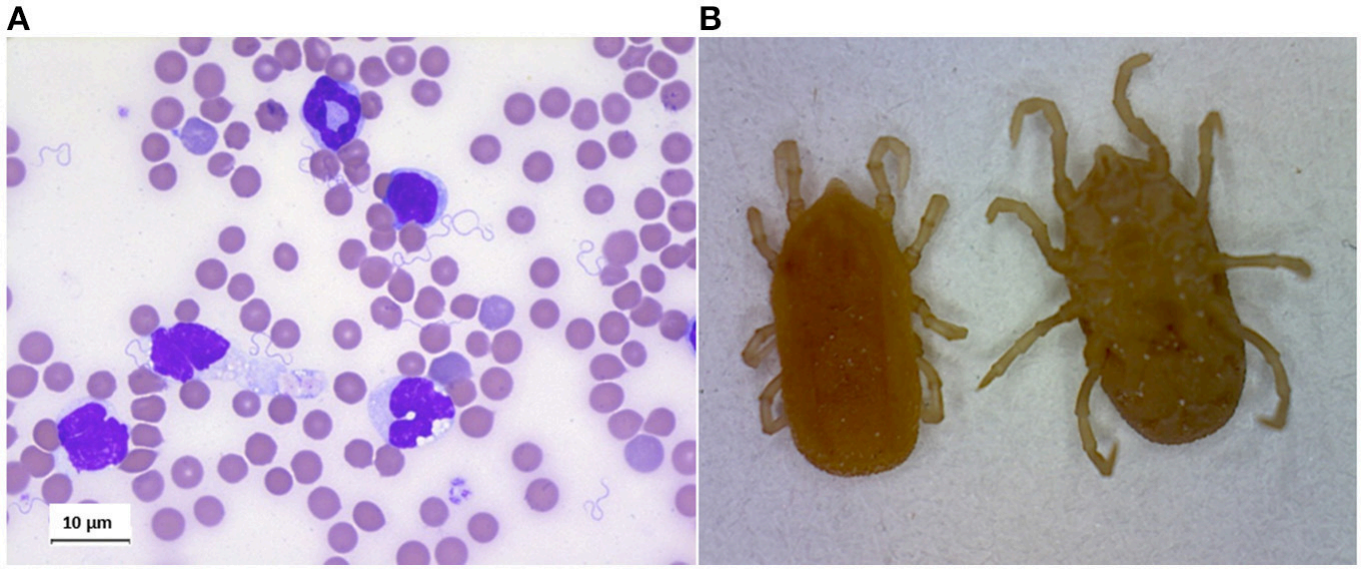
**(A)** Giemsa staing of blood smear of mouse infected by *Borrelia crocidurae*. **(B)** *Ornithodoros erraticus* adult, dorsal, and ventral views.

RF borreliae multiply in the circulation, where they can lead to very high spirochaetemia (Figure 4A). Besides, some of the Old-World RF *Borrelia* species, eg. *B. duttonii, B. crocidurae, B. persica* and *B. hispanica* (but not *B. recurrentis*), frequently interact with erythrocytes, causing them to aggregate, a phenomenon called erythrocyte rosetting. Rosetting was early observed by Mooser (1958), and *in vitro* models later suggested that this interaction is a way for the spirochaete to cover itself to escape the immune response. *B. crocidurae* which easily makes rosetting with erythrocytes, has a longer duration of the initial spirochetemia and also a delayed antibody response when compared to the non-rosetting strain *B. hermsii*, indicating that this may be one of the advantages of this strategy (Burman et al., 1998). Another hypothesis concerning the erythrocyte interaction is that the spirochaete picks up nutrients from the cells. In the murine model of *B. duttonii* infection, Larsson et al. have calculated as many as one billion bacteria in every milliliter of blood (Larsson et al., 2006), and obviously a lot of nutrients are needed to maintain the growth of such a population. This grazing theory is supported by the finding that the RF borreliae, in contrast to Lyme disease borreliellae, contain genes for utilization of purines as substrates for RNA and DNA synthesis (Pettersson et al., 2007). Since purine hypoxanthine is produced by red blood cells and abundant in human plasma, the purpose of the interaction with these cells is likely to supply *Borrelia* growth with this and other metabolites (Pettersson et al., 2007). These genes are missing in Lyme disease borreliellae which exceptionally reach such high density in the blood. As anemia and low concentration of erythrocytes are typical features of RF, spirochaete rosetting is likely to affect the red blood cells, as well as other cells in the circulation. Then this might cause a premature removal of affected cells. However, this theory has not been proven yet. Then again, thrombocytopenia, which also a typical consequence of RF borreliosis, is probably caused by spirochaete-platelet interactions such as *B. hermsii* attachment to platelets, resulting in increased platelet loss and prolonged bleeding. The depth of the thrombocytopenia was also linked to the degree of spirochetemia (Alugupalli et al., 2003b).

Finally, RF borreliae interactions with cells in the circulation might be a strategy to increase and prolong the time the pathogen can be maintained within the host. In addition, erythrocyte rosettes can protect the spirochaetes from the host immune defense and the subsequent loss of platelets will facilitate the penetration process into distant tissues.

## Tick-*Borrelia* interactions

### Vector specificity

The classification of RF borreliae was historically based on the concept of the specific relationship of arthropod-spirochete, meaning that a given bacterial species was carried by a particular vector and assuming a co-speciation (Wang and Schwartz, 2011). Numerous studies using gene sequencing have confirmed the genotype association both for RF borreliae strains and vectors [e.g., *B. crocidurae-O. sonrai*, (Trape et al., 2013); *B. turicatae-O. turicata* (Schwan et al., 2005)]. Several epidemiological studies have reported other tick-spirochaete associations which are in contradiction to the above vector specificity paradigm. As examples from soft ticks collected in North Africa, *B. crocidurae* DNA was detected in *O. erraticus* (Bouattour et al., 2010), DNA from *B. hispanica* in *O. marocanus, O. occidentalis* and *O. kairouanensis*, and *B. merionesi* DNA in *O. costalis* (Trape et al., 2013). Furthermore, some of the genomic species of RF borreliae share their vector with other *Borrelia* species (e.g., *B. microti* and *O. erraticus*; Naddaf et al., 2012). In some cases, the vector is even still unknown (e.g., *Candidatus B. algerica*; Fotso Fotso et al., 2015). However, studies reporting RF borreliae DNA in tick should be interpreted with caution given the fact that DNA traces found in a tick are not necessarily synonymous with vector competence. The proof of concept for vector specificity lies in the vector competence assays (Kahl et al., 2002).

For genomically distant species like *B. duttonii* and *B. anserina*, the transmission was not efficient by exchange of their respective natural *O. moubata* and *Argas* sp. ticks (Nicolle et al., 1928; Felsenfeld, 1971). Regarding closely-related RF borreliae, the cross transmissions of the North American *B. hermsii, B. turicatae* and *B. parkeri* have not been experimentally proven into *Ornithodoros* specimens other than their natural vectors *O. hermsi, O. turicata* and *O. parkeri*, respectively (Barbour and Hayes, 1986). However, *B. crocidurae* could be efficiently transmitted via a *O. erraticus* blood meal on rodents (Gaber et al., 1984), which is a natural vector of the other African species *B. hispanica* and “*B. microti*.”

### Tick midgut and salivary gland environments

During the multi-host development, soft ticks pass the usual three tick stages: larva, nymphs and male and female adults. While hard ticks have a unique nymphal stage, several nymph stages are present in soft ticks; they may pass through six or more nymphal stages, and females feed several times. After each blood meal they proceed for oviposition (Mehlhorn, 2001). The complete life cycle may last 20 years with prolonged periods of starvation. *Ornithodoros* ticks are also characterized by a quick engorgement, completing the blood meal within five to 60 min after host attachment, generally at night (Sonenshine and Roe, 2013).

As described previously, the STBRF borreliae comprise two main clusters: (1) The New-World TBRF borreliae, and (2) the Old-World TBRF borreliae (Table 1). At least 21 different species of STBRF Borreliae have been identified, associated with their own specific tick vector (Schwan and Piesman, 2002). Specific mechanisms of vector competence have evolved between species of RF borreliae and *Ornithodoros* ticks (Figure 4B).

The RF borreliae transmission by soft ticks is characterized by inoculation of saliva during the infective blood meal but also by the secretion of a pathogen-containing liquid from the coxal glands. However, only little work has been done so far to well understand the potential role of soft tick saliva in this process, while many more studies have been done in the hard *Ixodes* ticks, the vector responsible for the transmission of Lyme disease spirochaetes (Hovius et al., 2008; Kazimírová and Štibrániová, 2013; Liu and Bonnet, 2014).

#### Soft tick saliva

The first study performed on *Ornithodoros* saliva demonstrated the presence of antihemostatic activity and of apyrase (Ribeiro et al., 1991). Later on, thanks to the progress in proteomics and transcriptomic techniques, a few more investigations on soft tick saliva were published (Mans et al., 2002; Oleaga et al., 2007; Francischetti et al., 2008, 2009).

As described for hard ticks (*Ixodidae*), argasid saliva supports the feeding process by providing a cocktail of anti-hemostatic, anti-inflammatory and immunomodulatory molecules. The salivary transcriptome of the soft tick *Ornithodoros parkeri* refers to the presence of genes of the lipocalin family, as well as of several genes containing Kunitz domains indicative of serine protease inhibitors. Novel protein families with sequence homology to the insulin growth factor-binding protein (prostacyclin-stimulating factor), adrenomedulin, serum amyloid A protein precursor were characterized in soft ticks. The sialotranscriptome of *O. parkeri* confirms that gene duplication process is a common event in blood-feeding arthropods. Numerous homologies were found with the transcriptome of ixodid ticks (Francischetti et al., 2008). Using proteomic techniques (ED-gels and mass spectrometry analysis), molecules isolated from tick saliva of O. *moubata* and *O. erraticus* were characterized although not as efficiently as with the transcriptomic technique (Oleaga et al., 2007).

#### Soft tick midgut

The midgut of the tick is the first interface encountered by the bacteria during an infective blood meal. Therefore, proteomics studies have also been made on midgut tissue, allowing the identification of concealed antigens in the midgut which might serve as potential candidates for an anti-tick vaccine (Manzano-Román et al., 2006).

In a recently conducted thorough proteome study on *O. moubata*, the main vector of *B. duttonii* in Eastern and Southern Africa, a comparison between fed and unfed midguts was made. Interestingly, it revealed similarities between the blood digestion in hard ticks and in soft ticks (Oleaga et al., 2017). This study completed an earlier investigation on the transcriptome of *O. erraticus*, the vector of relapsing fever in South-Europe and also in Africa (Oleaga et al., 2015).

### Bacteria inside the tick

#### Soft ticks-borrelia interactions

Involvement of soft ticks in RF transmission was first described in Africa by Livingstone during his explorations in West-Africa as early as 1857 for *B. duttonii* transmitted by *O. moubata*. Then, Dutton and Todd published in 1905 the detection of a systemic infection of soft ticks with spirochaetes affecting the midgut, synganglion, malpighian tubules, salivary glands, ovary and coxal organs. Interestingly, a Burgdorfer's study in 1951, showed that the transmission occurred not only via tick bite but also by contamination with infected coxal fluid (Dutton and Todd, 1905; Burgdorfer, 1951). In addition, the mode of spirochaete transmission was different in the various tick stages: while the nymph transmits the bacteria with the saliva, the adults mainly transmit via the coxal fluid (Schwan and Piesman, 2002). These works also showed the initial midgut colonization, followed by the migration and the colonization of the salivary glands a few weeks later. Thus, in contrast to infected *Ixodes* where Lyme borreliellae persist in the gut only, RF bacteria infect the tick midgut in unfed ticks, and disseminate to other sites including salivary glands (Schwan and Piesman, 2002).

In the United States, three main species of *Borrelia* are found: *B. turicatae, B. hermsii* and *B. parkeri* transmitted respectively by *O. turicata, O. hermsii* and *O. parkeri* (Lopez et al., 2016). In Eurasia, the highest infection risk has been identified on the Iberian Peninsula and in Minor Asia (Rebaudet and Parola, 2006). The three main borreliae species there are: *B. hispanica, B. crocidurae*, and *B. duttonii* transmitted by *O. erraticus, O sonrai* and *O. moubata* respectively (Table 1). However, most of the studies conducted on the interaction tick-*Borrelia* have been accomplished in American models of TBRF.

#### B. hermsii

It is the primary cause of tick-borne relapsing fever in North America. *B. hermsii* is mainly contracted in remote areas of the Western United States and of British Columbia (Canada). The typical tick habitats are forested mountains at altitudes above 900 m. The main reservoir is rodents (Ogden et al., 2014). To investigate the interaction between *B. hermsii* and *O. hermsii*, a number of significant studies have been conducted by Schwan and collaborators. Looking first into the natural reservoirs, chipmunks and tree squirrels inhabiting coniferous forests, they demonstrated a rapid blood meal of 15–90 min occurring at night. The infected ticks were shown to keep the pathogens for years and to constitute real reservoirs in nature (Schwan and Piesman, 2002). All tick stages, the different nymph stages and the adults, transmitted the pathogen, although transovarial transmission was rare. Coxal transmission does not exist for this *Borrelia* species: *B. hermsii* is only transmitted by tick bite. However, systemic tick infection was demonstrated with these borreliae. An antigenic variation occurring during the process of transmission from the vertebrate host to the tick, was clearly described. The synthesis of a variable tick protein (Vtp formerly Vsp33 or Vmp33) occurs, likely under the influence of environment changes within the tick (pH, temperature and bacteria density) (Schwan and Hinnebusch, 1998). Like OspC for Lyme borreliosis spirochaetes (Grimm et al., 2004; Tilly et al., 2007), Vtp seems to be essential for the transmission to the vertebrate host. However, Vtp is expressed on all the bacteria in the tick, since in RF the transmission takes place within minutes. While the spirochaetes inside the tick vector express one unique protein, Vtp, the latter spirochaetes found in the blood of infected animals are able to express a multitude of variable major proteins (Vmps).

More precisely, in *B. hermsii*, Vtp production is higher in the spirochaetal population of the salivary glands than in midgut spirochaetes which express mainly Vmp. The proportion of Vtp+ spirochaetes from the salivary glands reaches 50% 35 days post infection and 90% 116 days post infection. A Δ*vtp* mutant of *B. hermsii* remains able to colonize *O. hermsi* but Vtp is not produced at the surface of spirochaetes during the vector phase and the tick-borne transmission is lost. Thereby, Vtp has an essential role in the *B. hermsii* tick-borne transmission (Raffel et al., 2014).

In *B. hermsii*, the interaction of spirochaetes, depleted in variable major proteins (Vmp) with specific antibodies, led to the identification of another tick protein, Alp (BHA128) (Marcsisin et al., 2012). This protein, identified by mass spectrometry, seems to be specifically expressed in the tick since it was produced at a higher level at 23°C than at 34°C. It is strongly expressed in tick salivary glands and expressed at a very low level in the blood of infected animals. Unlike Alp, *vtp* genes of *B. hermsii* share a high diversity among field spirochaetal population and are likely involved in horizontal genomic transfer and recombination events between strains (Porcella et al., 2005). This may be in part explained by a pathogen strategy influencing Vtp variability to avoid the vertebrate immune memory against previous transmitted Vtp+ spirochaetes (Marcsisin et al., 2016).

In this same model, *B. hermsii*–*O. hermsi*, a vaccine was tested using the Vtp antigen (Krajacich et al., 2015). The *vtp* gene from two isolates of *B. hermsii* was cloned and expressed as recombinant Vtps to vaccinate mice. Mice were protected only if they were challenged by *O. hermsi* ticks that were infected with the homologous strain of *B. hermsii* from which the *vtp* gene originated. Such a vaccine points out the difficulty to set up a protective vaccine against bacteria with such a high-protein diversity.

#### B. turicatae

This *Borrelia* is transmitted by *O. turicata* and present in the southern United States and in Latin America. Risks for exposure to *O. turicata* and *O. parkeri* occur primarily in semi-arid plains. *O. turicata* are parasites of ground dwelling and burrowing animals including tortoises (Adeyeye and Butler, 1989; Donaldson et al., 2016). In a mouse model used to investigate the process of transmission, the authors analyzed the dissemination in the blood by qPCR, dark field microscopy and serological responses. The transmission was found to occur within a minute, and dissemination into the blood was also very rapid. Inside the tick, *B. turicatae* entered the midgut and invaded the salivary glands during the following weeks (Boyle et al., 2014). This is clearly distinct from the LD borreliellae that remain in the midgut only. The blood meal on the vertebrate host triggers the migration of Lyme spirochaetes toward the salivary glands due to physico-chemical changes (pH, temperature, nutrients) (Schwan et al., 1995; Piesman et al., 2001; Schwan and Piesman, 2002). A more recent study by the same group completed these data, using the recent technology of the green fluorescent protein (gfp). It confirmed the systemic infection of ticks and also a persistent infection of the tick midgut and salivary glands for at least 18 months (Krishnavajhala et al., 2017). The spirochaetes were shown to be maintained transstadially, i.e., during six or more nymphal stages before molting to adults. After blood feeding on the vertebrate host, the salivary gland lumen of infected ticks remained positive. This indicates that the *Borrelia* inoculum is probably low, as described for Lyme borreliosis spirochaetes (Kern et al., 2011; Bockenstedt et al., 2014). The midgut remains positive after the blood meal as well. This midgut population likely replenishes the salivary glands after the infective blood meal. It also explains the rapid transmission of bacteria to the vertebrate host during the timely short blood meal of soft ticks (Krishnavajhala et al., 2017).

Using microarrays on *B. turicatae* grown *in vitro* at 22°C, thus mimicking the tick environment, a protein of 40 kilodaltons was identified and designated *Borrelia* repeat protein A (BrpA) due to the repetition of a particular amino acid motif. Deletion of the respective *brpA* gene did neither avoid the infection of mice when inoculated by needle, nor inhibit further colonization of the *O. turicata* salivary glands and the subsequent transmission (Lopez et al., 2013).

Finally, it has been postulated that the tick salivary glands might constitute a selective environment for a particular *Borrelia* species. Indeed, in different tick species (*O. hermsi, O. parkeri*, and *O. turicata*) engorged on mice infected with *Borrelia hermsii*, only the association *O. hermsi*-*B. hermsii* was able to further transmit *Borrelia* to naïve mice (Schwan, 1996). *Borrelia*-gfp should help to understand the mechanisms responsible for the specificity of the interactions vector-pathogen (Krishnavajhala et al., 2017).

## Hard tick-transmitted relapsing fevers

Although the majority of RF spirochaetes infections occur through soft ticks, few spirochaetes species are transmitted by hard ticks. This includes *B. theileri, B. miyamotoi* and *B. lonestari* which are transmitted by *Rhipicephalus* spp., *Ixodes* spp. and *Amblyomma americanum* ticks, respectively (Table 1). Very few studies have been conducted on these systems, likely because their pathogenicity to humans is not clearly established except for *B. miyamotoi* (Platonov et al., 2011). Considering all RF borreliae, a relative specificity can be postulated since the phylogenetically close species *B. miyamotoi, B. theileri*, and “*B. lonestari*” are associated with hard tick as vectors, while others are transmitted by soft ticks (*Argas* spp. for *B. anserina* or *Ornithodoros* spp.). Interestingly, newly described genotypes close to “*B. lonestari*/*B. theileri*” have been detected in hard ticks in Japan (*Haemaphysalis* sp. or *Amblyomma* sp.) and in Ethiopia (*Rhipicephalus* sp.) (Takano et al., 2012; Kumsa et al., 2015; Furuno et al., 2017), but “*Ca*. B. texasensis” close to the North American *B. parkeri*/*B. turicatae* according to *flaB* and *rrs* sequences is associated with the dog hard tick *Dermacentor variabilis* rather than an *Ornithodoros* soft tick (Lin et al., 2005).

### Borrelia miyamotoi

Although considered as worldwide species, the evolution of *B. miyamotoi* may also have been under the influence of its global distribution because this species is represented by the Siberian, European and American genotypes (*glpQ*, 16S rDNA, and/or *flaB. SLPA*) (Mun et al., 2006; Crowder et al., 2014; Takano et al., 2014). Geller and collaborators (Geller et al., 2012) have shown that the Asian genotype of *B. miyamotoi* could be associated as well with *I. ricinus* as with *I. persulcatus* ticks in a sympatric region of Estonia. In addition, *B. miyamotoi* genotypes are characterized by a very low diversity within an area, and even considered as genetically clonal isolates after MLSA whatever the sources *I. persulcatus, I. pavlovskyi* ticks or vertebrates in Hokkaido, Japan (Takano et al., 2014).

#### Reservoir

There is little information concerning *B. miyamotoi* reservoirs, as this bacterium shares common characteristics with the bacteria belonging to the *B. burgdorferi* sl complex, it can be easily hypothesized that they share the same reservoir hosts. Small rodents such as the white-footed mouse (*Peromyscus leucopus*) in North America (Bunikis and Barbour, 2005; Barbour et al., 2009; Hamer et al., 2012); *Apodemus argenteus* (Fukunaga et al., 1995), *Apodemus speciosus, Myodes rufocanus, Myodes rutilus* (Taylor et al., 2013) in Japan; *Apodemus flavicollis* (Szekeres et al., 2015; Hamšíková et al., 2017), *Myodes glareolus* (Hamšíková et al., 2017; Wagemakers et al., 2017), *Apodemus sylvaticus, Microtus arvalis* (Wagemakers et al., 2017) have been found to harbor *B. miyamotoi* DNA. Identically *B. miyamotoi* DNA has been found in birds like *Meleagris gallopavo* (Scott et al., 2010), *Carduelis chloris* and *Parus major* (Wagemakers et al., 2017). Only *Apodemus* spp. mice, *M. glareolus* (Burri et al., 2014) and *P. leucopus* (Scoles et al., 2001) are experimentally proven reservoir hosts, indeed *B. miyamotoi* horizontal transmission to naïve ticks has been observed. However, there is a lower rate of transmission than the one noticed for the *B. burgdorferi* sl complex bacteria. It has also been observed that *B. miyamotoi* infection rate of wild rodents is age-independent whereas *B. burgdorferi* sl infection rate is age-dependent, these data suggest that *B. miyamotoi* is shortly maintained by the small rodents (Taylor et al., 2013). Conversely to what is observed for the *B. burgdorferi* sl complex bacteria, domestic ruminants do not seem to eliminate *B. miyamotoi* (Richter and Matuschka, 2010) and may play a role in its dissemination.

#### Interaction vertebrate host bacteria

Until 2011, pathogenicity of *B. miyamotoi* was unknown. Interest in this bacterium has grown up since 2011 when a series of 46 Russian cases were published (Platonov et al., 2011). Its pathogenicity was then precise with the report of two meningoencephalitis cases in highly immunocompromised patients (Gugliotta et al., 2013; Hovius et al., 2013), an additional case was later reported in Germany (Boden et al., 2016). The three cases of *B. miyamotoi* meningoencephalitis seem to display common characteristics, indeed they were treated for non-Hodgkin lymphoma with a CHOP protocol (cyclophosphamide, doxorubicin, vincristine and prednisolone), they also received an anti-CD20 monoclonal antibody (rituximab) which is known to deplete B-cell lymphocytes. All 3 cases of *B. miyamotoi* presented a cerebrospinal fluid pleocytosis with an elevation of proteins as observed for the Lyme neuroborreliosis. Clinically there is a disparity between the German case who had acute symptoms (Boden et al., 2016) and the other two (Gugliotta et al., 2013; Hovius et al., 2013) who evolved on a more chronic mode. These reports highlight that *B. miyamotoi* has a neurotropism whose physiopathology remains unknown.

Data describing the typical *B. miyamotoi* disease (BMD) in apparently immunocompetent patients come from 2 series of cases from Russia (Platonov et al., 2011) and from the United States (Molloy et al., 2015). The most reported clinical form is an acute febrile viral-like illness occurring around 2 weeks after the tick bite which can evolve toward one or more relapses (Platonov et al., 2011) for 11% of patients in the Russian cohort. In addition to fever, the symptoms include chills, headache, myalgia, arthralgia, malaise and fatigue. BMD and human anaplasmosis share common clinical characteristics and must be the subject of a differential diagnosis (Chowdri et al., 2013; Molloy et al., 2015). *B. miyamotoi* broadens the circle of the aetiological agents responsible for post-tick bite febrile syndromes.

The study of the physiopathology of diseases relies on experimental models and *in vivo* studies allow a global approach of the host/bacterial interactions. There are few data on the animal model for *B. miyamotoi* infection. It has been shown that SCID mice develop a prolonged spirochaetemia (up to 20 days) following an intra-peritoneal injection of *B. miyamotoi* culture (Krause et al., 2015; Wagemakers et al., 2016). C3H/HeN mice develop a peak in spirochaetemia 2 days after an intraperitoneal injection, followed by spirochaetemic relapse of a lower intensity in three out of the eight C3H/HeN mice used for the experiment (Wagemakers et al., 2016).

Concerning the interaction of *B. miyamotoi* with the host immune system, like other pathogens responsible for STBRF (*B. parkeri, B. duttonii*, and *B. hermsii*), *B. miyamotoi* is resistant *in vitro* to the human complement whereas *B. anserina*, a non-pathogenic STBRF for humans, is sensitive to the human complement (Teegler et al., 2014; Wagemakers et al., 2014). It has been observed that the activated complement components (i.g. C3, C5, C7, C8, C9, and the membrane attack complex) poorly or do not bind to *B. miyamotoi* under *in vitro* conditions, suggesting that the inhibition of the complement cascade occurs at the C3 activation level (Teegler et al., 2014). This mechanism was initially poorly understood since a protein assimilated to OspE, identified in *B. miyamotoi*, is unable to bind to factor H (McDowell et al., 2003). Recently, a protein factor CbiA (complement binding and inhibitory protein A) (Röttgerding et al., 2017) has been identified as binding and inhibiting the human complement at different levels. CbiA is a *B. miyamotoi* outer surface protein which binds to the complement regulators factor H and C4b-binding protein. Moreover, factor H bound to CbiA can interact with factor I to inactive C3b. Additionally, CbiA binds to C3, C3b, C4b, and C5 in a dose dependent manner. Finally, CbiA inhibits activation of the classical and terminal complement pathway.

### Borrelia lonestari

#### Reservoir

*Borrelia lonestari* reservoir host is not clearly identified. As the white-tailed deer (*Odocoileus virginiatus*) is a reservoir host for several *A. americanum*-associated pathogens (Allan et al., 2010) it can be easily hypothesized that the white-tailed deer could be a reservoir for *B. lonestari* in nature. Moreover, it has been shown to develop a bacteraemia following a *B. lonestari* injection (Moyer et al., 2006) and *B. lonestari* was detected in its blood compartment (Moore et al., 2003). However, serological studies supporting contact between *O. virginiatus* and *B. lonestari* are contradictory (Krishnavajhala et al., 2017). Interestingly, the DNA of a *Borrelia* species very close to *B. lonestari* was detected in *C. nippon yesoensis* (Lee et al., 2014) suggesting that cervids could be, indeed, reservoir hosts for *B. lonestari* and closely related species. Other observations mentioned that *B. lonestari* DNA was found in several birds particularly in turkeys (Jordan et al., 2009). Further investigations must be led to better understand the ecology of *B. lonestari*.

#### Interaction vertebrate host bacteria

*Borrelia lonestari* is found in *Amblyoma americanum* also known as the Lone Star tick and gave its name to this relapsing fever *Borrelia*. Initially researchers hypothesized that the southern tick-associated rash illness (STARI) was caused by *B. lonestari*. Indeed, patients frequently bitten by *A. americanum* developed symptoms similar to erythema migrans observed in Lyme disease (Armstrong et al., 1996; Kirkland et al., 1997). The parallel between erythema migrans/*B. burgdorferi* sl bacteria, and, STARI/*B. lonestari* can easily be done and *B. lonestari* DNA was found in the skin biopsy of a patient with a STARI (James et al., 2001). However, since its discovery (Lin et al., 2005) *B. lonestari* implication in the STARI has been discussed. More recent observations (Wormser et al., 2005; Feder et al., 2011) demonstrate that STARI occurs without proof of *B. lonestari* implication. So far, there is no proof of *B. lonestari* implication in human or animal pathology.

### Borrelia theileri

#### Reservoir

*Borrelia theileri* has been observed in the blood compartment of cattle (Laveran, 1903), sheep (Theiler, 1905), and in a horse (Callow, 1967). However, horses had previously been reported as non-susceptible to this infection (Theiler, 1905). The cattle can be considered as the main reservoir host for *B. theileri* since it has been demonstrated that the cattle can infect naïve ticks (Theiler, 1909; Brumpt, 1919; Trees, 1978) and since *B. theileri* is vectored by ticks belonging to the genus *Rhipicephalus* (*R. annulatus, R. microplus, R. decoloratus, R. evertsi*) which preferentially parasites the cattle (Guglielmone et al., 2014).

#### Interaction vertebrate host bacteria

To our knowledge there is no report of human infection by *B. theileri*. Its pathogenicity is expressed in cattle but remains low (Callow, 1967; Smith et al., 1978). Work by L. L. Callow reports a transitory rise of the rectal temperature and occasionally a slight depression with anorexia and hemoglobin level decrease has been observed in a splenectomized calf (Callow, 1967).

## Conclusion

Compared to Lyme borreliosis, RF diseases remain poorly investigated. The phylogeny of RF bacteria still deserves further investigation to better understand the complex interactions *Borrelia*-tick-vertebrate host and establish adapted models. It is particularly true for bacteria transmitted by soft ticks which represent most of the bacteria in RF. For example, the soft tick saliva is not so well studied. While numerous saliva tick proteins have been characterized in hard ticks and demonstrated essential in pathogen transmission, very few ones have been identified in *Ornithodoros* ticks. Similarly, the process of transmission and persistence of RF bacteria in vertebrate host is not clarified, although antigenic variations and erythrocytes rosetting have been described as potential virulence factors. The emergence of *B. miyamotoi* transmitted by hard ticks these last years might draw more attention to these diseases which are present in tropical as well as temperate countries.

## Author contributions

ET-R, PB, SB, LV, and NB conducted the literature research, wrote the paper and prepared the figures and tables. All authors provided critical reviews and revisions.

### Conflict of interest statement

The authors declare that the research was conducted in the absence of any commercial or financial relationships that could be construed as a potential conflict of interest.
